# Exploring magical thinking in the context of pain

**DOI:** 10.3389/fpain.2026.1753496

**Published:** 2026-07-23

**Authors:** Birgit Heckemann, Lionel Snell, Mark I. Johnson

**Affiliations:** 1 Department of Anaesthesiology and Intensive Care Medicine, Sahlgrenska University Hospital/Östra, Gothenburg, Sweden; 2 Institute of Health and Care Sciences, University of Gothenburg, Gothenburg, Sweden; 3 Independent Book Author and Practitioner, Cape Town, South Africa; 4 Centre for Pain Research, School of Health, Leeds Beckett University, Leeds, United Kingdom

**Keywords:** chronic pain, coping strategies, illness narrative, magical thinking, meaning-making, mind body connection, patient experience, subjective experience of pain

## Abstract

This perspectives article explores magical thinking (which is often dismissed as irrational or pathologized) as a potentially valuable personal orientation and resource for people living with chronic pain: as a way to make more sense of their complex and challenging life experience, psychologically as well as socially. *Magical thinking* entails a broader acceptance of connectivity between phenomena generally considered to be independent, and it includes symbolic *meaning-making acts* such as rituals. The authors explicitly exclude in their discussion *magical superstitions* which involve the conviction that magical acts can directly impact physical reality (such as enabling levitation or turning people into frogs). Drawing on a reflective, interpretive, and conversational methodology, a pain scientist, a nurse researcher, and a writer-practitioner of magical thinking explored how magical thinking functions as a personal orientation and resource that enables symbolic meaning-making and supports emotional regulation and relation with self, others and the wider world. As such, it may be potentially valuable for people living with chronic pain. The article shows that traces of magical thinking can be found within psychology and medicine and invites a broader conversation about how the symbolic and imaginative modes of magical thinking might be valuable in pain care.

## 1 Introduction

This article explores and discusses magical thinking in the context of chronic pain. While few publications address this topic, a recent article highlights the relevance of magical thinking (which is often dismissed or pathologized) to evidence-based practice medical care (1). Magical thinking, like religion and spirituality (2) or artistic expression (3), may help people make sense of the complex and often challenging experience of living with chronic pain. While religion and art have received some scholarly attention in pain research (2, 3), magical thinking^1^ remains largely marginalized and excluded from the scholarly pain discourse.

## 2 Aim

This article offers an exploration, discussion and an invitation to consider magical thinking as a potentially supportive orientation for people living with chronic pain.

## 3 Approach: interpretive conversational inquiry

We adopted a reflective, interpretive, and conversational approach grounded in life- and practice-based experience to co-construct conceptual insight rather than generating empirical generalisations.

We conducted a series of online conversations via Microsoft Teams, organised in dyads and triads (see Table 1).

**Table 1:** Conversations, topics and participants.

Online conversation #	Topic	Participants	Date
1	An initial conversation focusing on the integration of magical thinking into everyday life and its relevance for pain.	BH, MIJ	2025 04 08
2	Exploration of shared interests, decision and verbal agreement to co-author a manuscript based on a conversation between BH and LS.	BH, MIJ, LS	2025 04 11
3	In-depth exploration of the topic through a conversation.	BH, LS	2025 05 08
4-6	Discussions of key insights.	MIJ, BH	2025 06 10 2025 07 17 2025 10 04

Conversation 3 between BH and LS which explored LS's viewpoints on magical thinking^2^ was recorded and auto-transcribed. BH and MIJ identified key insights from the conversation. All authors discussed the key insights based on a purposeful (rather than systematic) search of databases (PubMed and Google Scholar). They selected scholarly sources for their conceptual relevance.

Author position statements are provided in the Supplementary Material.

## 4 Exploring magical thinking through conversation and scholarship

This section presents the key insights from Conversation 3 and discusses them in relation to relevant scholarly literature.

### 4.1 Conversation starter 1: what does magical thinking mean to you personally?

#### 4.1.1 Key insight: magical thinking offers a sense of agency and empowerment in responding to life's challenges

LS shared stories from a higher-level perspective, drawing on his lived experience and long-standing engagement with magical practice. He explained how people use symbolic action^3^ to try and make things happen or achieve a goal in situations where learned knowledge does not help. LS illustrates this by recounting a story about young men who think that attracting a female partner is a problem that can be solved by knowledge acquisition. They study psychology, read self-help books, and try to understand “what women want” (LS). Yet they are not successfully attracting women. Frustrated, they turn to buying clothes, such as a T-shirt with a logo from a fashionable brand, convinced by adverts that wearing this brand will make them more attractive. And indeed, LS continued, wearing the T-shirt makes the students feel more confident, they act differently, and suddenly they are more able to connect with women. For LS, this T-shirt acts as a symbolic object, a modern talisman^4^. While the T-shirt itself has no power, it enables a transformation from failure to success through symbolic action. For LS, this example captures how symbolic action can restore a sense of agency when reason and knowledge fall short. He suggests that magic often begins at the point where people seek power through symbolic means when rational strategies and reason fail.

### 4.2 Conversation starter 2: how would you define magical thinking?

#### 4.2.1 Key insight: magical thinking invites understanding rather than definition

**Figure 1:**
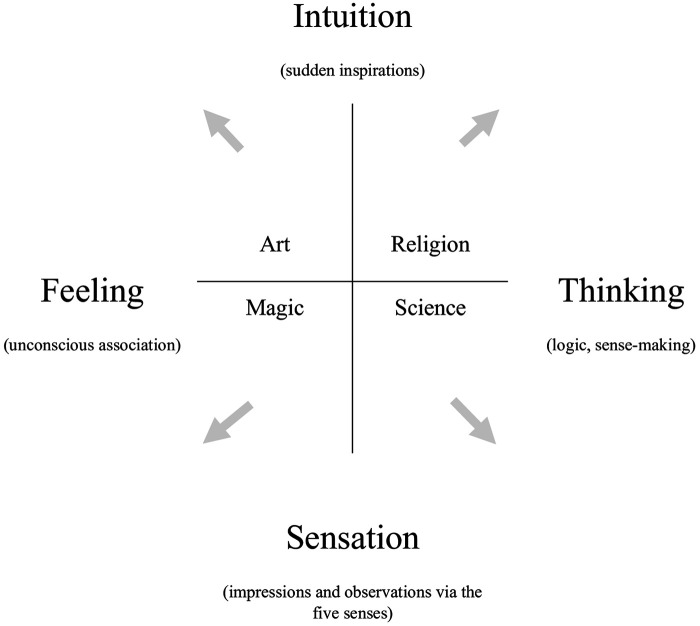
Mapping the intersection between art, science, religion, and magic across psychological Jungian functions. Reproduced and adapted with permission from the original creator and co-author L.S. [Snell (26), p. 125].

LS explained that he prefers not to define magical thinking but rather understands it as a way of engaging with the world, an orientation. LS considers magical thinking as a discrete category but an approach to life that is about searching and discovering. LS mapped the relationship between the four disciplines art, science, religion and magic in relation to the Jungian psychological functions thinking and feeling, intuition and sensation in his published work (5, 26) (Figure 1). However, a map is never the territory, and Figure 1 illustrates that the disciplines are orientations rather than defined categories, and overlaps may occur between the fields.^5^

To illustrate this, he returns to the example of the young university students who would dismiss a magical thinking approach to attracting women as nonsense. Yet they slip into magical thinking. Wearing the “latest trendy things,” LS observed, can change how a person feels and how others respond, producing effects that are psychologically real even if not empirically causal or residing in the object. For LS, magical thinking is therefore not opposed to science or psychology, but reflected in both disciplines, through symbolic action and meaning making. Thus, magical thinking is not a fixed or discrete belief system, but a context-dependent engagement mode that people move in and out of, often without explicit acknowledgement. This understanding of magical thinking aligns with broader anthropological and psychological perspectives that regard magical thinking as an ordinary, situational and everyday human behaviour rather than a practice or a belief limited to specific groups or individuals.^6^

### 4.3 Conversation starter 3: how do you use magical thinking to support your wellbeing or manage pain?

#### 4.3.1 Key insight: magical thinking as a natural human capacity that creates meaning

In our conversation, LS described magical thinking as a natural human trait. He explained how magical thinking is present throughout life, from infancy to adulthood. He described how babies first discover the physical world through repetition, for example dropping a spoon, watching it fall, and thus learning that some things (the spoon always falls down) are predictable. This early experimentation, which he called a form of “protoscience,” marks the beginning of causal understanding. Yet when the mother's reactions vary, sometimes picking up the spoon, sometimes not, the child begins to notice that not everything follows fixed rules. The mother's behaviour introduces unpredictability, prompting the child to infer the existence of other minds, and the wish to communicate with these minds. From this insight grows the capacity to perceive agency beyond the self, not only in people but also in animals, plants, the weather, or even objects. For LS, this transition from materialist certainty (the spoon always falls) to relational curiosity (how can I make mama pick up the spoon?) represents “the beginning of magical thinking,” the recognition that “minds could exist outside your own head.”

LS's reflections exemplify how magical thinking can mature into a reflective and relational worldview. He describes moving from a youthful, instrumental approach “making things happen” to dialogue and participation. Everyday objects become personalised rather than being inanimate tools: he speaks to his car keys and thus remembers where he put them. He also explains how behavioural change can happen through personalising objects. Regarding one's running shoes as partners to establish a fitness routine supports building new habits and continuity. This approach can be likened to the currently popular “life hacking”^7^, but he highlighted that the relationship between the object and the person goes deeper, that indeed an ongoing social interaction, like a partnership, with an object might occur. He explained that when using magical thinking “[…] you’re part of this bigger universe”, a world is animated, meaningful, and responsive.

### 4.4 Conversation starter 4: why do you turn to magical thinking to support wellbeing or manage pain?

#### 4.4.1 Key insight: magical thinking embraces ritual and wonder and creates resilience

In our conversation, LS described how the health-related value of magical thinking lies less in measurable outcomes than in orientation. It fosters a stance of resilience and curiosity that transforms misfortune into engagement rather than despair. As he explained:

“A lot of people I know are much more vulnerable to moods, depressions… I’m more resilient. I think it's [magical thinking] contributed to that. When I have a run of bad luck […] I’m as much curious as to what's happening as I’m miserable about the round of bad luck.” (LS)

LS describes a shift from emotional reactivity toward a curious, reflective stance. From this perspective, personal or cultural events are interpreted as part of larger patterns or cycles, rather than as isolated threats to the individual. This perspective supports resilience and coping by creating psychological distance and meaning. A similar stance can be observed in religious contexts, for example when hardship is framed as “God is testing my faith” (LS). LS also described magical thinking as enriching life through seasonal and celebratory rituals, less instrumental than expressive, as a way of attuning to nature. For him, it simply “makes life more interesting.” (LS), by opening a space where mystery and imagination coexist with everyday reality. The world becomes populated with subtle energies, spirits, and presences “things that cannot be seen with the eye” (LS) yet shape what happens around us. If flowers bloom unusually well, one might attribute it to “flower spirits” (LS) rather than analyse soil or weather conditions. LS' openness cultivates a world that is alive, relational and dynamic and never entirely understood.

### 4.5 Conversation starter 5: when do you use magical thinking to support your wellbeing or manage pain?

#### 4.5.1 Key insight: magical thinking as communion between mind and body

LS described using magical thinking to relate to recurring pain or distress rather than eliminate it. Drawing on his book *The Little Book of Demons* (7), he likens pain to an unwelcome but familiar visitor “a boring neighbour who keeps coming round” (LS). Rather than “killing the messenger,” he might talk to the pain and asks about its message:

“Pain, why are you here? Why are you in my arm? What do you want?” “Are you asking me to stop gym workouts? For ever? Or how long?” Maybe the pain suggests that you stop barbell curls for a while to allow your arm to heal. Every few days you might check: “Do you still want me to stop doing exercise? Is it working? Are you healing?” And the pain says “Yes” (LS).

He explains that these answers are not expected to arrive as spoken language. Instead, while focusing on the sensation of pain, the individual might rehearse various alternatives in imagination—such as changing their diet, massaging the area, increasing exercise intensity, or bypassing certain movements—to sense which scenario invokes a feeling of relief. This approach, he suggests, transforms pain from an enemy into an advisor, part of an ongoing relationship with the body. In this reframing, pain is personified—not as a hostile force to be fought, but as a familiar presence to be acknowledged, negotiated with, and even befriended. Magical thinking thus becomes a form of embodied dialogue that restores agency, curiosity, and acceptance in the face of discomfort.

LS suggests that both religious and magical thinking offer frameworks of meaning and orientation toward forces perceived as larger than the individual, which can transform the pain experience. Whereas religious believers may frame suffering as “God is testing me”, LS personally adopts a more animistic stance, treating the body itself as a wise, ancient entity shaped by millions of years of evolution and capable of considerable self-healing. By asking his [personified] pain “to explain what it is doing,” (LS) he engages his body as a partner rather than an object to be managed, LS describes his approach to managing health and pain as guided by instinct, moderation, and trust in natural processes. He identifies as a naturopath by inclination, preferring to minimise medication and observe the body's response before escalating treatment after surgery:

“If they [healthcare professionals] say, if you don't take it [pain medication] today, tomorrow you’ll be in agony, OK, I’ll take [one] because I trust them––but I won't take three just to see what happens.” (LS)

## 5 Discussion

In this section, we discuss these key insights in relation to scholarly literature before relating magical thinking and chronic pain.

### 5.1 Agency and empowerment

LS' view of magical thinking as fostering agency and empowerment echoes anthropological accounts that see it as a pragmatic response to uncertainty. Malinowski (1979) argued that magical thinking is not an irrational practice but an adaptive strategy that enables action and motivation when rational strategies fail (8). This description resembles how Indigenous peoples engage with the world, as described in anthropological literature on animism and relational ontology (9, 10), a worldview grounded in reciprocal coexistence with land, animals, and even inanimate objects (9). From this non-human-centric vantage point, magical thinking becomes a collaborative, non-dominant way of relating to the world.

### 5.2 Understanding

In contrast to LS' description, scholarly literature often *defines* magical thinking as “the belief that thoughts, actions, or symbols possess the power to cause, change, or prevent real-world events” (1: Iserson 2023, p. 132). Magical thinking ususally makes no sense within the current paradigm of science as it relies on subjective experiences and the assumption that imperceptible forces cause real-world effects” (6). Although LS rejects this definition^8^, both views agree that magical thinking is not irrational but an alternative orientation that creates meaning, detects patterns, or offers practical ways to handle existential and other challenges, serving social, cognitive, emotional, or adaptive functions (6).

### 5.3 A natural human capacity

LS's account of how magical thinking develops aligns with Piaget (11) who regarded magical thinking as a stage of childhood cognition that adults outgrow, and more recent literature which suggests that magical thinking persists and transforms across the lifespan (4). As children mature, scientific reasoning strengthens, but symbolic and imaginative sense-making remains and may be expressed through artistic or everyday rituals in later life.

According to LS, magical thinking makes the world a more exciting place. Likewise, Subbotsky (2010) argues that, for adults as well as children, magical thinking brings colour and excitement to everyday life, counteracts monotony, and rekindles a sense of wonder. The desire for magic explains the enduring appeal of magical narratives such as *The Lord of the Rings* or *Harry Potter* (4).

### 5.4 Ritual, wonder and resilience

LS considers himself more resilient than many other people when experiencing a streak of bad luck. As a magically thinking person, he considers himself part of a bigger universe and can simultaneously feel miserable and curious about his situation. This capacity has been described as self-distancing (12), which has been shown to be effective to facilitate emotion regulation in challenging situations (13).

Seasonal, social or private rituals help structure experience, mark transitions and create coherence amid uncertainty (14). They foster a sense of belonging and wonder. This sense of mystery and connection parallels the experience of awe and wonder that literature links to wellbeing and flourishing (14, 15). Rituals, however, need not involve magical thinking; secular or therapeutic practices such as mindfulness can retain symbolic functions without any magical connotations (16).

### 5.5 Communion between body and mind

LS describes a strong attunement between mind and body, seeing them as communicating rather than separate. By “talking” to the painful parts, he can selectively follow medical advice in ways that maintain coherence and preserve his sense of agency despite the pain. The literature shows that magical thinking can give a sense of control over illness. This “illusion of control” may reduce anxiety, support a more adaptive mindset, and shape stress and meaning-making processes that influence how pain is experienced (17).

### 5.6 Magical thinking and chronic pain

Even within broadly dualistic Western traditions, pain is increasingly understood as a complex and relational experience that exceeds the Biopsychosocial (BPS) model, which is necessary but not sufficient to capture how pain is lived and managed over time (18). In addition to biological, psychological, and social factors, pain is shaped by ecological processes: the material, institutional, and environmental conditions within which people act, including healthcare systems, work and domestic arrangements, cultural practices, and technologies of care. These ecologies structure concrete actions and practices, such as: navigating fragmented services, self-monitoring symptoms, experimenting with treatments, avoiding or ritualising daily activities, and developing personalised routines to manage uncertainty. Within such contexts, practices sometimes described as “magical thinking” can be understood not as irrational belief, but as situated attempts to establish causal coherence, restore a sense of agency, or stabilise meaning when reliable explanations or effective interventions are lacking. While the BPS model guides chronic pain treatment, it is often applied in practice as a checklist, privileging biomedical factors while under-addressing these broader contextual dynamics (19). Several contemporary models of chronic pain (particularly enactive, embodied, and contextual approaches) foreground relational and meaning-making dimensions that align with the concerns outlined here.

Karos et al. (2018) conceptualized pain as a complex social experience that “shifts control away for the person with pain to others, […] excludes, and […] is often associated with (perceived) injustice”, p. 1692 (20). As a consequence, chronic pain threatens a person's needs for autonomy, belonging, justice and fairness (20). An orientation towards magical thinking may support a person feeling more empowered and as a part of “a bigger universe” (LS), and, as a result, more resilient and less helpless.

Another recent perspective, the integral model of pain, extends the BPS model by positioning pain as a relational, context-sensitive, and embedded experience in a whole person, health and wider system (21). Consistent with salutogenic thinking, this perspective shifts attention from dysfunction towards the cultivation of health, meaning, and coherence. A sense of coherence rests on three interrelated components: comprehensibility (structured and predictable life challenges), manageability (belief in coping resources), and meaningfulness (life's demands are worth engaging with) (22). While key insights *agency and empowermen*t align with the manageability component, the magical thinking orientation (with its non-rational assumptions) stands in tension with comprehensibility and meaningfulness: with a magical-thinking orientation, a person can easier cope with challenging situations. LS illustrates this with his description of a streak of bad luck, where one feels miserable but curious at the same time. This ability to be distressed while also being a curious observer has been shown to decrease the intensity of acute pain sensations (23).

The concept of painogenic environments highlights how cultural, organisational, and socio-ecological conditions, contribute to sustaining pain and undermining resilience by shaping how people experience, interpret, and respond to pain over time (24). Painogenicity encompasses both indirect conditions that increase the likelihood that pain will persist once present and, in some cases, upstream exposures that directly cause diseases in which pain is an intrinsic feature. What unifies these influences is not their mechanism of action but their ecological role: they shape the background conditions within which pain emerges, persists, and becomes difficult to resolve. Some painogenic factors act indirectly by undermining recovery, increasing regulatory strain, or restricting adaptive responses, while others exert more direct biological effects through tissue damage or disease processes. Painogenicity therefore functions as an ecological lens rather than a claim about causation per se, highlighting how diverse influences, direct and indirect, interact to sustain enduring patterns of pain.

From this perspective, pain is not only physiological but relational, and managing it may require reconnecting with social contexts, everyday environments, and opportunities for meaningful participation (25). Taken together, the integral, salutogenic, and painogenic perspectives echo Indigenous ontologies that emphasise relationality between individuals, communities, and environments (9, 10), and resonates with what LS describes as a “magical thinking” orientation, in which the self is understood as part of a “bigger universe”, a metaphor for embeddedness within wider relational and ecological worlds.

## 6 Limitations

This perspectives article is not based on rigorous empirical research but aims to open a space for discussion. We sought to corroborate our key insights with relevant literature. Although confirmation bias cannot be ruled out, we addressed this by engaging with diverse sources and considering counter-positions where possible.

## 7 Conclusion

This perspectives article, grounded in an interpretive dialogical inquiry, invites consideration of magical thinking as a potentially supportive orientation for people living with chronic pain. Although imagination and symbolic meaning-making are central to human experience, they are often marginalised within dominant Western ontologies. Recognising their role may offer new insights into patients' lived experiences, not by treating magical thinking as a testable belief or therapeutic intervention, but as a situated mode of meaning-making that shapes how pain is understood, endured, and managed.

From a research perspective, magical thinking can be explored using established qualitative and interpretive approaches including: narrative and phenomenological studies of pain experience, ethnographic observation of everyday coping practices and rituals, participatory and co-design methods, and comparative analyses of pain models that foreground relational and symbolic dimensions. Such approaches do not seek to validate or promote specific beliefs, but to understand how symbolic and imaginative forms of meaning-making operate within broader ecologies of care.

The clinical relevance of this article lies in its balanced analysis of magical thinking, framing it as a significant and deeply human trait that carries both potential and risk. Despite its prevalence, magical thinking is currently stigmatised and dismissed within Western biomedical approaches to pain and illness treatment. Stigmatising these beliefs encourages patient non-disclosure, which obscures the identification of potential risks associated with magical thinking and prevents the integration of adaptive magical-thinking coping strategies into treatment. When symbolic or culturally embedded forms of meaning-making are marginalised in healthcare encounters, patients may disengage from care, selectively follow medical advice, or turn away from Western models altogether. Conversely, unexamined magical thinking may contribute to selective adherence or the substitution of symbolic practices for essential treatments. These tensions highlight the need for an interpretive and dialogical approach that engages with patients' meaning-making while maintaining a commitment to safety, evidence, and shared decision-making.

In this way, alternative ontologies, including forms of magical thinking, may complement biopsychosocial models by illuminating how people make sense of pain as a multifaceted, relational phenomenon, without requiring the rejection of biomedical care. However, as the practical integration of these divergent worldviews remains underexamined, further research is needed to explore how clinicians might engage with symbolic meaning-making while upholding safety and evidence-based standards. Qualitative methodologies offer valuable frameworks to explore and illuminate how magical thinking can safely coexist with, and potentially even enhance, current medical approaches to pain treatment.

## Data Availability

The raw material can be made available upon reasonable request.

## Footnotes

^1^At this junction, it is important, however, to distinguish between *magical thinking* and *magical beliefs (superstition)* Magical thinking entails the conviction that symbolic associations, rituals, or intentions can influence real-life outcomes, independent of empirical causality. Magical beliefs (superstition) is an extension of magical thinking that entails the conviction that magical entities, forces can directly impact or transform physical realities without any causal link (such as turning lead into gold) (4). This article is exclusively concerned with *magical thinking* and seeks to contribute to the scientific discourse by integrating insights from authorial dialogue and interdisciplinary literature, including anthropology, psychology, and religious studies to challenge prevailing assumptions.

^2^Authors BH and MIJ developed conversation starters that guided conversation 3:

1. What does magical thinking mean to you personally?
2. How would you define magical thinking?
3. How do you use magical thinking to support your wellbeing or manage pain?
4. Why do you turn to magical thinking to support wellbeing or manage pain?

^3^Symbolic action is the use of symbols, gestures, rituals, or language to convey a meaning or value that extends beyond the literal or practical utility of the act itself. It may be compared with artistic expression.

^4^A talisman is an object that is thought to have magic powers and to bring good luck. Reference: https://languages.oup.com/google-dictionary-en/

^5^Psychology as a science, for example, may work with anchors such as visualization, suggestion or images which are techniques that can also be found in magic, art and religion.

^6^It is necessary to distinguish LS's descriptions from the scholarly definition of magical thinking: the belief that “thoughts, actions or symbols possess the power to cause, change, or prevent real-world events” and that “thoughts can exert physical influence” (6, p. 132). While we acknowledge the conceptual and cultural breadth of this definition, our focus remains on the practical application of magical thinking—specifically, how individuals mobilize it to manage pain when conventional biomedical reasoning feels insufficient. Our position does not endorse supernatural causality; rather, we aim to understand how symbolic and imaginative practices provide meaning, coherence, and agency.

^7^A strategy or technique adopted to manage one's time and daily activities in a more efficient way.

^8^LS argues that "magical thinking" is not merely a belief, but a form of personal experimentation based on trial and error. He distinguishes between three frameworks: science (objective testing), magic (subjective experience), and religion (communal identity). While science and magic both grow through open observation, LS defines religion as a community held together by shared beliefs and the rejection of dissent. He illustrates this by critiquing the secular "sceptics" movement of the early 21st century. Although these groups claimed to defend science by campaigning (for example) to ban homeopathy, LS suggests their behaviour was actually religious. By forming a community based on institutional prohibition and the denial of alternative views—rather than on open experimental debate—they adopted the dogmatic structures they intended to oppose.
