# Supplementary material for: Exploring magical thinking in the context of pain
Source: Front Pain Res (Lausanne). 2026 Jul 23;7:1753496. doi: 10.3389/fpain.2026.1753496 (PMC13442387; doi:10.3389/fpain.2026.1753496)
Supplement: Supplementary file 1 [file Datasheet1.docx]

Supplementary Material

# **Philosophical and Cultural Foundations of the Authors**

# **Birgit Heckemann**

# Associate Professor Birgit Heckemann is a registered nurse and qualitative researcher focusing on the topics of chronic pain, person-centred care, patient and public involvement, and digital health. Birgit regards chronic pain as a multi-dimensional expression of physical or mental trauma that affects all areas of life. Birgit is also a certified embodiment coach. Her interest in magical thinking is rooted in her work as a trauma-informed embodiment coach, where she supports clients in deep body-based explorations to process past experiences and enable positive change. These explorations are often experienced as deeply healing and described as *magical*. These experiences triggered Birgit’s interest in exploring magical thinking. Epistemologically, Birgit is rooted in a constructivist realist tradition, regarding knowledge as shaped by human interpretation, yet grounded in an external reality. She works with qualitative narrative, descriptive, and interpretive research methodologies and mixed methods research.

# **Lionel Snell**

# Lionel Snell studied mathematics at Clifton College, Bristol and Cambridge University, two institutions with significant magic/occult libraries. There he noted the similarity between magical and mathematical thinking. Whereas scientific thinking must focus on what can be proven to exist, and religious thinking must focus on what can be believed, magic and pure mathematics can freely explore what is irrational or not real (the magician Austin Spare prescribed acting “as if” something were true).

# Thus liberated from the religious and scientific constraints of Absolute Reality, Lionel wrote (under the pseudonym Ramsey Dukes) *SSOTBME An Essay On Magic* (1976)*,* since reissued, translated into Polish, German and Spanish, and widely recognized (along with 9 further books) as a significant influence on the Chaos Magick current. He has been a member of occult organisations including OTO and IOT, he has given public lectures on Magic in England, Austria and South Africa and is best known for his Ramsey Dukes YouTube channel exploring magical thinking. Lionel’s most recent book is *My Years of Magical Thinking*(2017).

# **Mark I. Johnson**

# Professor Mark I. Johnson trained as a physiologist and pain scientist and adopts a critical realist stance, blending his positivist scientific background and decades of research experience with a commitment to methodological rigour, reflexivity, and inclusivity, acknowledging both objective reality and the shaping role of sociocultural contexts. His theoretical approach to chronic pain is grounded in a culturally informed, integrative model, drawing from Integral Theory, biopsychosocial and salutogenic paradigms, and evolutionary perspectives. Within this framework, pain is conceived as a complex, multi-layered phenomenon shaped by biological, psychological, cultural, and spiritual dimensions, while also recognized as an emergent property of the nervous system embedded in the living body. Rather than reducing pain to linear cause-effect mechanisms, this perspective emphasizes its dynamic, relational nature—arising from interactions among neural processes, bodily states, and sociocultural contexts. Moving beyond the traditional biopsychosocial model, pain is situated within a broader framework of embodied cognition and meaning-making, where neurophysiological activity is inseparable from lived experience and environmental affordances.
